# Case of Pleuro-Tuberculosis

**Published:** 1856-07

**Authors:** F. B. Norcom

**Affiliations:** Chicago, Ill.


ARTICLE III.—*Case of Pleuro-Tuberculosis.* By F. B. Norcom, M.D., Chicago, Ill., May, 1856.

The following case is an abstract, taken from the history book of the Third Medical Division of Bellevue Hospital. There is very little alteration from the original, and the statements are founded upon carefully observed facts, rather than upon vague recollections.

Ward 49, bed 680: James Britt, æt. 34 years; tailor by occupation; Irishman, resident of New York city for last six years; and admitted May 17th, 1855.

Several circumstances in his hereditary history, point to the existence of a predisposition to phthisis. His mother and her brother died of pleurisy, (as he calls it,) after a protracted illness. He is now the only surviving member of the family, his two sisters having fallen victims to a disease which, from its symptoms, strongly indicates a tubercular character.

With the exception of two severe attacks of fever, also one of an intermittent type, his previous health remained perfectly good, until he left home at the age of 18 years to find employment in cities; he then became exceedingly irregular in his habits, indulging freely in alcoholic potations and venereal excesses. About this period, contracted a chancre, followed by buboes; and, a while afterwards, suffered from secondary and tertiary rheumatic pains. Was salivated during the treatment for his specific disease. After his removal to this country, he wholly abstained from spirituous liquors for 2½ years; but, his health beginning to fail, he resumed their use, and has been intemperate ever since. His habits and occupation expose him to frequent colds, which, however, seldom persist long enough to give much inconvenience. Never had hæmoptysis.

His present illness dates from the 1st of May, and he ascribes it to a cold taken during exposure. He was first seized with darting pains in the left side of chest anteriorly, accompanied with difficulty of respiration, a dry cough, and some little febrile reaction. On admission, a physical examination was made: a slight bulging of left side, though no well-marked dilitation of intercostal spaces; vocal vibration entirely lost, and expansile movements limited. Percussion gives dulness, rising to the middle of scapula; absence of respiratory murmur, and faint ægophonic resonance at surface of fluid. Percussion sounds of right side normal, and the supplementary respiration as usual in such cases. Heart pushed to right of the sternum, irregular in its contractions, and impulse feeble. Decubitus upon the diseased side. His general condition exhibited great weakness and prostration; pulse running over a hundred, small and fluttering; respiration hurried.

Ordered—℞: Potass. acetatis., as a diuretic; vegetable tonics, with iron internally, and blisters to the side.

This plan of treatment was persevered in for three weeks, without any marked improvement in his general condition, or any perceptible diminution of the effusion. It was then changed—the pil. triplex, tonics, nutritious diet, and flying blisters, but with no better success. He failed day by day, suffering considerably from dyspnœa and irregular shooting pains in the chest, to which was now superadded hectic fever, anorexia, and sleeplessness.

On the 12th June, a consultation was called, and the visiting physician, Prof. Metcalfe, deemed it advisable to perform the operation of paracentesis thoracis, empyema being strongly suspected. His condition upon this day was as follows: Pulse 96; resp. 32; skin hot and moist: (signs as before noted—effusion to about middle of scapula, heart beating to right of sternum, and puerile respiration on right side, with absence of vocal respiratory sounds on left.)

Cough and dyspnœa, excited by movement or pressure upon diaphragm. Measurement of thorax gave the following dimensions:—

Around the nipple, (R. S.,) 18¼ inches.

" " " (L. S.,) 18½ inches.

Around lower extremity of sternum, (R. S.,) 18 inches.

" " " " (L. S.,) 17¼ inches.

An opening was made in the seventh intercostal space, of the left lateral region, by a small exploring trocar, followed by a larger instrument, the patient lying on his back; 96 oz. of pale limpid straw-colored serum, flowed freely through the canula, affording almost immediately a sense of great relief. The lips of aperture were then kept apart by a small piece of lead wire doubled upon itself, inserted in the opening, its ends being bent and secured on the outside by strips of adhesive plaster.

Ordered—℞: Ammon. bi-carb., grs. v., "ter in die," as a diuretic, together with tonics and good diet.

13th.—Feels more comfortable; can now lie on his back; skin moist; no increase of dulness, and faint respiratory murmur heard.

19th.—Pulse 80; resp. 24 and easy; skin hot and moist. Phys. examination indicated a re-accumulation of the fluid. Pulsation of heart still felt to right of sternum, and decubitus entirely dorsal. Percussion anteriorly resonant to edge of axilla, where it becomes flat; dulness posteriorly extends upward to region of scapula. Respiration puerile on right side, weak respiration and broncophony on left. Operation repeated, the fluid ran very slowly, and only 6 oz. could be obtained with difficulty by suction, of the same color and density as the first drawn. Ordered—Spts. ætheris nitrici, ℨi., "ter in die," &c.

20th.—Pulse 80; resp. 24; skin hot and dry; has felt chilly occasionally through the day; cough not troublesome, nor excited by pressure on diaphragm; decubitus dorsal—though can lie on either side.

21st.—Patient passed a restless night, perspiring very freely; urine scanty, not albuminous.

25th.—Paroxysms of hectic continue to recur; slight increase in diuresis. A physical examination, made this morning, revealed the increase of fluid to its former level. Faint œgophony at surface. About 11 A.M., was removed to an upper and better ventilated ward. In afternoon, pneumothorax detected; the time of perforation not marked by any acute symptoms. ℞: Quinæ. sulphat., grs. v.; morphiæ sulphat., gr. $\frac{1}{4}$.; M. et fiat chart; to be taken at night.

28th.—Felt more comfortable, but is evidently losing ground. Physical signs demonstrate circumscribed pneumothorax posteriorly—metallic tinkling, &c., &c. From this date, the patient began to fail rapidly, refusing to rally under the most stimulating and nutritious diet. Died, July 17th.

*Sectio Cadaveris*—Tuesday, July 17th, 2 P.M., 8 hours after death.—Rigor mortis well marked; body pale and much emaciated; no signs of decomposition, though weather intensely warm.

*Thorax.*—On raising the sternum, slight adhesions were found posteriorly to right lung, by recently effused false membrane; left lung adherent anteriorly to the portion of sternum covering it; left border of heart in the median line of the body. Ninety-six ounces of greenish serum distended the pleural sac, the upper stratum of which was transparent, whilst the lower turbid and purulent. On inserting a tube in trachea and inflating the lungs, the right became easily distended, while the left admitted none, being much compressed, and reduced to not more than one-quarter its natural size, lying against anterior medi astinum, occupying the upper portion of the pleural cavity and extending to the diaphragm by a thin prolongation. The whole of the left pleura covered by a thick false membrane. Right lung crepitant, normal in size and appearance, structure healthy: upper portion of left, hard and carnified, containing numerous small, yellow tuberculous deposits; the remaining portion very slightly crepitant and free from disease. Remaining viscera healthy.

I was not before aware of the imperfections of this record; it is certainly to be regretted, yet I much prefer giving it as copied from my notes, than attempting to supply the deficiencies by stimulating the memory. This case is one, occasionally met with in practice, occurring as a complication of Phthisis in about 10 per cent.,* and dependent upon tuberculous deposits in the lungs. It may be regarded as a sub-acute variety of pleurisy, and is met with, we believe, invariably in persons of a scrofulous diathesis.

The diagnosis made from the first physical examination, was simply pleurisy with effusion: and though from the hereditary history of the patient, we had strong grounds to suspect the presence of tuberculous trouble; yet the symptoms, both physical and subjective, were not sufficiently well-marked to demonstrate its existence. It was only after we had watched the progress of the disease for some time, and recognized not only its persistence but the inutility of our therapeutical agents to subdue it, as also the progressive emaciation and prostration of the patient, that our presumption amounted almost to a certainty.

The late Prof. Swett, did not consider that, in such cases, there was much of a tendency to the formation of pus; and though empyema was strongly suspected from the general condition of the invalid, still there was only a serous or sero-albuminous fluid obtained. The lead wire was left in the wound until a re-accumulation became apparent—then removed—not one untoward symptom having followed this plan.

This idea was introduced into the Hospital by Prof. Metcalfe, who, at that time, employed it in his private practice.

---

* M. Louis' Summary of Path. Anat. of Phthisis.

